# Nitric Oxide Production in the Striatum and Cerebellum of a Rat Model of Preterm Global Perinatal Asphyxia

**DOI:** 10.1007/s12640-017-9700-6

**Published:** 2017-01-21

**Authors:** M. Barkhuizen, W. D. J. Van de Berg, J. De Vente, C. E. Blanco, A. W. D. Gavilanes, H. W. M. Steinbusch

**Affiliations:** 1 Department Pediatrics, School for Mental Health and Neuroscience (MHeNs), Maastricht University, Maastricht, The Netherlands; 2 Department Psychiatry and Neuropsychology, School for Mental Health and Neuroscience (MHeNs), Maastricht University, Maastricht, The Netherlands; 3 EURON - European Graduate School of Neuroscience, Maastricht, The Netherlands; 4 DST/NWU Preclinical Drug Development Platform, North-West University, Potchefstroom, South Africa; 5 Department of Anatomy and Neurosciences, Neuroscience Campus Amsterdam, VU University Medical Centre, Amsterdam, Netherlands; 6 Institute of Biomedicine, Faculty of Medicine, Catholic University of Guayaquil, Guayaquil, Ecuador; 7 Department of Translational Neuroscience, Faculty of Health, Medicine and Life Sciences, Maastricht University, P.O. Box 5800, 6212 AZ Maastricht, The Netherlands

**Keywords:** Asphyxia, Nitrosidative stress, cGMP, Neuronal nitric oxide synthase, Peroxynitrite, Selective vulnerability

## Abstract

Encephalopathy due to perinatal asphyxia (PA) is a major cause of neonatal morbidity and mortality in the period around birth. Preterm infants are especially at risk for cognitive, attention and motor impairments. Therapy for this subgroup is limited to supportive care, and new targets are thus urgently needed. Post-asphyxic excitotoxicity is partially mediated by excessive nitric oxide (NO) release. The aims of this study were to determine the timing and distribution of nitric oxide (NO) production after global PA in brain areas involved in motor regulation and coordination. This study focused on the rat striatum and cerebellum, as these areas also affect cognition or attention, in addition to their central role in motor control. NO/peroxynitrite levels were determined empirically with a fluorescent marker on postnatal days P5, P8 and P12. The distributions of neuronal NO synthase (nNOS), cyclic guanosine monophosphate (cGMP), astroglia and caspase-3 were determined with immunohistochemistry. Apoptosis was additionally assessed by measuring caspase-3-like activity from P2-P15. On P5 and P8, increased intensity of NO-associated fluorescence and cGMP immunoreactivity after PA was apparent in the striatum, but not in the cerebellum. No changes in nNOS immunoreactivity or astrocytes were observed. Modest changes in caspase-3-activity were observed between groups, but the overall time course of apoptosis over the first 11 days of life was similar between PA and controls. Altogether, these data suggest that PA increases NO/peroxynitrite levels during the first week after birth within the striatum, but not within the cerebellum, without marked astrogliosis. Therapeutic benefits of interventions that reduce endogenous NO production would likely be greater during this time frame.

**Electronic supplementary material:**

The online version of this article (doi:10.1007/s12640-017-9700-6) contains supplementary material, which is available to authorized users.

## Introduction

Neonatal encephalopathy (NE) due to perinatal asphyxia (PA) is a common cause of morbidity and mortality in the period around birth. In 2010, 8.5 infants per 1000 live term-births developed PA-related encephalopathy (Lee et al. 2013). The long-term neurological outcome after NE varies, from normal neurocognitive functioning after mild asphyxia to cerebral palsy, epilepsy, cognitive, behavioural or memory problems in severe cases (Armstrong-Wells et al. 2010; Volpe 2012). PA-related injury to the preterm brain is even more complex, since prematurity by itself increases the risk of NE (Volpe 2009a). While preterm birth before 37 weeks of gestation occurs in 5–8% of all pregnancies, very low gestational age (VLGA) birth, before 32 weeks of gestation occurs, in about 1% of singletons and 9% of twin pregnancies (Schaaf et al. 2011). In cohorts of preterm infants with multifactorial encephalopathy, behavioural, cognitive, attention or social deficits have been reported in 25–50% of cases and 5–10% had major motor impairments. A large portion of this disability burden was made up by VLGA infants, due to improved survival rates with modern medical care. Although the major motor impairments are striking, cognitive deficits are far more common (Volpe 2009a; Volpe 2009b). In preterm infants, PA causes both white matter injury of the developing oligodendrocytes (periventricular leukomalacia) in the sub-cortical regions and associated grey matter injury to the striatum and other basal ganglia structures, thalamus, basis pontis, brain stem and cerebellum (Cabaj et al. 2012; Logitharajah et al. 2009; Shah et al. 2006). Injury in these infants is a combination of primary destruction after PA and secondary maturational and trophic disease (Volpe 2009a).

PA occurs when oxygen supply between the mother and the foetus is disrupted, causing a biphasic brain injury. The acute injury results from the combined effects of cellular energy failure, acidosis, glutamate release, intracellular calcium accumulation, lipid peroxidation and nitric oxide (NO) neurotoxicity that disturb vital cell components. This results in cell death. From 6 to 48 h after the insult, a secondary cerebral energy failure occurs with mitochondrial dysfunction due to sustained pathological reactions in the primary phase (Perlman 2006; Piña-Crespo et al. 2014). The time-window for therapeutic intervention is thus limited. Currently, therapeutic hypothermia is the only therapy available for term infants with NE. This only partially reduces morbidity, and it is not used in preterm infants (Edwards et al. 2010). There is thus an urgent need for therapies in the preterm- and term infants. Animal models are essential tools for the preclinical development and testing of new therapies. In humans and large animals, the majority of brain development occurs prenatally. However, in rodents, the brain is still immature at birth and only resembles the term human infant at roughly 7–10 days postnatal (Semple et al. 2013). This also makes the rat a suitable organism to study insults to the preterm brain. Our group, and others, have optimized a rat model of global anoxia during birth to investigate PA in the rodent equivalent of the 23–32-week-old human foetus (Semple et al. 2013).

In this study, we focused on two structures important for motor control and coordination, which are in differential developing states at the time of insult, namely the striatum and the cerebellum. The primary roles of the striatum involve learning of associations between stimuli, actions and rewards (Balleine et al. 2007), the selection between competing response alternatives and motivational modulation of motor behaviour (Lenz and Lobo 2013; Liljeholm and O’Doherty 2012). In humans, neurogenesis of the striatal medium spiny neurons begins around week 11.5 and striatal synaptogenesis begins around 13 weeks of gestation. Synaptogenesis is well-established before mid-gestation, with near-uniform synapses by 34 weeks of human gestation (Freeman et al. 1995; Sarnat et al. 2013). The cerebellum undergoes rapid expansion from week 24 to 40 of gestation. During this time, the cerebellar volume increases by 3.5–5-fold. The cerebellum is particularly vulnerable in preterm infants, due to its rapid growth towards the end of gestation (Volpe 2009b). The cortico-striato-cerebellar tract is instrumental for motor sequence learning (Tzvi et al. 2014). In addition to its established role in motor coordination, the cerebellum also directs linguistic and related cognitive and behavioral-affective functions (De Smet et al. 2013). Damage to these two regions is of clinical significance for both the motor deficits and cognitive/attention deficits after PA (Volpe 2009b; Volpe 2012).

The NO cascade has emerged as both a major player in neurotoxicity—and a potential therapeutic intervention in PA. During PA, NO is involved in both the early phase injury and during secondary energy failure. Immediately after, asphyxia at birth neuronal nitric oxide synthase (nNOS) is activated, increasing NO neurotransmission. After 12–24 h, inducible nitric oxide synthase (iNOS) is activated in glial cells which leads to cerebral NO production (Gunes et al. 2007; Perlman 2006). Concurrent increases in the generation of superoxide cause the formation of peroxynitrite (ONOO^−^). Peroxynitrite is a potent oxidative agent which causes tissue injury and contributes to ischemic injury in the immature brain, by irreversibly inhibiting the mitochondrial respiratory chain (Ikeno et al. 2000; Weis et al. 2011). The newborn brain is especially vulnerable to this type of insult due to its relatively low antioxidant levels (McQuillen and Ferriero 2004; Shim and Kim 2013). The extent of excessive NO production is dependent on the severity/duration of asphyxia, with variability across different neuron types and nervous system locations (Dorfman et al. 2009; Klawitter et al. 2007). Nitrotyrosine—a product of peroxynitrite and proteins—was present in the brain tissue of human NE infants at autopsy (Groenendaal et al. 2006). Moreover, increased NO in the cerebrospinal fluid in the first 24 h correlates with the severity of the NE (Ergenekon et al. 2004; Gunes et al. 2007). The exact role of NO and peroxynitrite in mediating the effects of PA remains to be elucidated. Pharmacological inhibition of neuronal nitric oxide synthase (nNOS) has been proposed as a strategy to reduce cerebral palsy and other motor deficits after PA (Ji et al. 2009; Yu et al. 2011) and nNOS inhibitor potent neuroprotective agents in most animal models for hypoxia-ischemia and excitotoxicity in vitro. Selective inhibition of nNOS shows beneficial effects including preservation of mitochondrial function after in utero ischemia. nNOS inhibition also improves the short-term survival of GABAergic interneurons in the striatum in hypoxic ischemic preterm sheep and delays the onset of post-asphyxial seizures (Drury et al. 2014; Rao et al. 2011). Inhaled NO has shown beneficial effects in rodent models of neonatal stroke. However, high doses of NO, or low doses of NO, administered during the reperfusion period are detrimental (Charriaut-Marlangue et al. 2012).

The aim of this study was to determine the timing and regional selectivity of increased NO production global PA in brain areas as striatum and cerebellum, involved in motor regulation and coordination structures. Targeting supraphysiological NO production could be a fertile therapeutic target for the preterm infant. In order to use NO production for a therapeutic target, we need to know at which time points after PA NO production peaks, whether these peaks coincide with increased cell death and whether regional differences exist in the brain.

## Materials and Methods

### Animals

Full-term pregnant Wistar rats (Charles River-Broekmans, Someren, The Netherlands) and their male pups (*n* = 108) were used for the present study. Female pups were sacrificed at birth. The pups were divided into two groups (PA and control). Six pups per group were used at each time-point (suppl. Fig. S1). They were housed under standard conditions (12:12 h light: dark cycles, 20°C) with free access to standard laboratory chow and water. The local Committee on Animal Welfare approved all animal care and procedures. Within this study, exclusively male offspring was used because both morphological and behavioural studies provided evidence for a differential vulnerability to a birth insult in males versus females. A greater impact is seen in the male sex, probably due to the protecting role of the circulating hormones in females (El-Khodor and Boksa 2003; Zhu et al. 2006).

### Induction of PA

PA was induced in rat pups at P0 by placing the uteri and its contents in a water bath for 20 min, as described previously (Vlassaks et al. 2014). Briefly, time-pregnant Wistar dams were decapitated immediately after delivery of two pups (control vaginal deliveries) and rapidly hysterectomized. The uterus horns containing the remaining pups were placed in a water bath at 37°C for 20 min (severe PA). Afterwards, pups were removed from the uterus horns and stimulated to breath by cleaning their skin and by gently padding them on the chest. The pups were left to recover for 60 min in a paediatric incubator at 36.5°C and randomly assigned to a surrogate mother (10 pups per mother), which had given birth on the same day. The percentage of mortality in the PA and control group was, respectively, ±50 and 0%.

### 4,5-Diaminofluorescein Diacetate (DAF-2/DA) Detection in Slices

For the DAF2/DA detection in fresh tissue, six rat pups were decapitated and their brains were rapidly removed and placed into ice-cold Krebs-Ringer bicarbonate buffer (pH 7.4) aerated with 95% O_2_/5% CO_2_. The forebrain and the cerebellum were chopped into 300-μm coronal slices using a McIllwain tissue chopper. Slices were separated under a microscope and transferred to a multi-well culture plate containing Krebs buffer (4°C; pH 7.4) and 1 mM isobutyl-methyl xanthine (IBMX) to inhibit 3,5′-cyclic nucleotide phosphodiesterase (PDE) activity. Alternated slices were transferred to a second multi-well plate and used for nNOS and cGMP immunohistochemistry. All slices were incubated in Krebs-Ringer buffer containing IBMX for 30 min and slowly warmed to 35.5 or 25.5°C under an atmosphere of 5% CO_2_/95% O_2_ prior to DAF2/DA staining.

DAF-2-triazole (DAF-2/T) fluorescence was studied in slices (300 μm) including the striatum of rat pups at postnatal day P5, P8 and P12 (*n* = 6 per group at each time-point) using the DAF2/DA detection assay (Sigma, The Netherlands) according to the method of Lopez-Figueroa et al. (2000). Upon entry into the cell, DAF-2/DA is hydrolysed by cytosolic esterases, producing DAF-2. DAF-2 reacts with NO and peroxynitrite to form the highly fluorescent derivative DAF-2/T (Bryan and Grisham 2007; Rodriguez et al. 2005; Roychowdhury et al. 2002). The slices were incubated with 1.5 ml 10 μM DAF-2/DA incubation buffer (150 mM Tris-HCl, 3 μM tetrahydrobiopterin, 1 μM flavin adenine dinucleotide (FAD, Sigma), 1 μM flavin mononucleotide (FMN, Sigma), 1 mM NADPH, 0.6 mM CaCl_2_ (Merck) and 100 μM L-arginine (Sigma) per well for 45 min at 35.5 or 25.5°C. During incubation with the DAF-2/DA solution, lights were turned off and a dark box was placed over the incubation chamber. Following incubation with DAF-2/DA, the slices were washed in phosphate-buffered saline (pH 7.4) and placed on non-coated slides and cover slipped using PBS-glycerol (1:4). To confirm that DAF-2/DA has a high affinity for NO, slices were incubated in the presence or absence of the NO donor 0.1 mM sodium nitroprusside (SNP; Sigma) for 10 min, or the NOS inhibitor 0.1 mM *N* ^*G*^-nitro-L-arginine (Sigma) for 30 min. As a negative control, slices were incubated in media lacking DAF-2/DA.

### nNOS, cGMP and Caspase-3 Immunohistochemistry

Slices of the striatum and cerebellum (*n* = 6, 10-μm thick sections adjacent to the slices used in the DAF-2/DA experiment) were used to visualize cGMP-producing and NOS active structures, using antisera against cGMP (de Vente et al. 1987) and nNOS (Herbison et al. 1996). After incubation in the presence or absence of SNP or *N* ^*G*^-nitro-L-arginine, slices were fixed for 2 h with 4% paraformaldehyde and cut into sections with a cryostat. The sheep anti-cGMP and anti-nNOS antisera were used, respectively, at a dilution of 1:4000 and 1:2000 and visualized using an Alexa-conjugated donkey anti-sheep antibody (1:100; Mol. Probes, USA). The nNOS and cGMP sections were processed to study co-localization with cell-specific markers. An antibody against glial fibrillary acidic protein (anti-GFAP) (1:1600; Sigma) was used to identify astrocytes (*n* = 5). The immunolabelling was visualized with a Cy3-conjugated donkey anti-mouse antiserum (1:800) or goat anti-mouse Alexa Fluor® 488 (1:100; Mol. Probes, USA). The sections were incubated overnight at 4°C with the primary antibody followed by 2 h at room temperature with the secondary antibody. Negative controls were performed by omitting the primary antibody. nNOS immunoreactivity labelled low-threshold spiking interneurons in the striatum and the cerebellar granule cells. Parvalbumin immunohistochemistry identified the fast-spiking striatal interneurons and cerebellar Purkinje cells. Basket and stellate interneurons in cerebellar molecular layer express both parvalbumin and nNOS (Contestabile 2012; Lenz and Lobo 2013; Schwaller et al. 2002). Parvalbumin-immunoreactivity (*n* = 6) was visualized with a rabbit antiserum (1:1500) provided by P.C. Emson (Babraham Institute, Cambridge, UK) in combination with a donkey anti-rabbit biotinylated antiserum (1:400) and streptavidine-Cy3 (1:2000). Sections were cover slipped using TBS: glycerol (1:3). Sections were examined at a magnification of ×400 and ×1000 with an Olympus AXE-70 microscope.

For caspase-3 immunohistochemistry, another set of six pups were anaesthetized with sodium pentobarbital and perfused transcardially with a fixative containing 4% paraformaldehyde and 2% picric acid. Afterwards, the brains were snap-frozen and cut into 16-μm thick coronal sections. These sections were incubated with a rabbit polyclonal anti-caspase-3 antibody (67341A; Pharmingen, Europe) diluted 1:500. The anti-caspase-3 antibody was visualized using a biotinylated goat anti-rabbit antibody (1:400; Jackson Immunoresearch Laboratories) and streptavidine Cy-3. Caspase-3 positive cells were counted in tissue of six rats of each group (PA and control) at P8 as described previously in Van de Berg et al. (2002). The total amount of caspase-3-positive cells was estimated by multiplying the number of counted cells in all sections by the sampling interval (i.e., equal to eight).

### Fluorometric Assay of Caspase-3-like Activity

Another set of six control and six asphyctic rat pups was used for analyses of caspase-3-like activity at P2, P5 or P8, P11 and P15 in cerebellum homogenates. The cerebellum was collected and homogenized in a lysis buffer containing 137 mM NaCl, 20 mM Tris-HCl (pH 8.0), 1% NP-40, 10% glycerol and a complete protease inhibitor tablet (Roche, NL). The tissue samples were briefly centrifuged and an aliquot of the supernatant (30 or 50 μl) was used. The assay is based on fluorometric determination of the cleavage of the Ac-Asp-Glu-Val-Asp-AMC peptide (Ac-DEVD-AMC; Biomol, Germany) by caspase-3 as described in detail previously (Van den Hove et al. 2006). The cleavage was followed at 2 min intervals for 3 h.

### Image Analysis

Fluorescence intensity of the images obtained from the slices loaded with DAF-2/DA and immunostained sections were analysed using a macro designed for measuring grey values in a given area (region of interest, ROI). Background values were measured using the slices incubated with the incubation buffer lacking the DAF-2/DA or lacking the primary antibody. Measurements were performed on three sampled images of three systematically sampled slices. From these data, the mean grey value in the area was calculated. Digital images were captured using a CoolView CCD camera system attached to a MR C600 confocal microscopy (Leica Microsystems, Germany). The microscope was equipped with a narrowband MNIBA-type FITC filter, or MNG filter for CY fluorescence (Chroma Technology, The Netherlands). Excitation was measured at 488 nm and emission at 530 nm. Grey scaled images were directly converted into artificial colours with the analySIS® image analysis system. All sections were stained simultaneously and recorded on the same day to minimize experimental variation.

### Statistics

Group comparison (controls vs. PA) of the number and caspase-3 positive cells as well as the grey values of immunofluorescence intensity measurements were analysed using the Student *t* test. The differences in Ac-DEVD-AMC cleavage between control and asphyctic rats were evaluated using a pairwise two-way ANOVA analysis and post hoc tests using Bonferroni correction for repeated measures. Statistics were carried out using the SigmaStat™ software version 2.03. Differences were considered significant if *P* ≤ 0.05. All data are presented as mean ± standard error of the mean (SEM).

## Results

### Timing of Regional NO Production in the Rat Asphyctic Striatum and Cerebellum

**Fig. 1:**
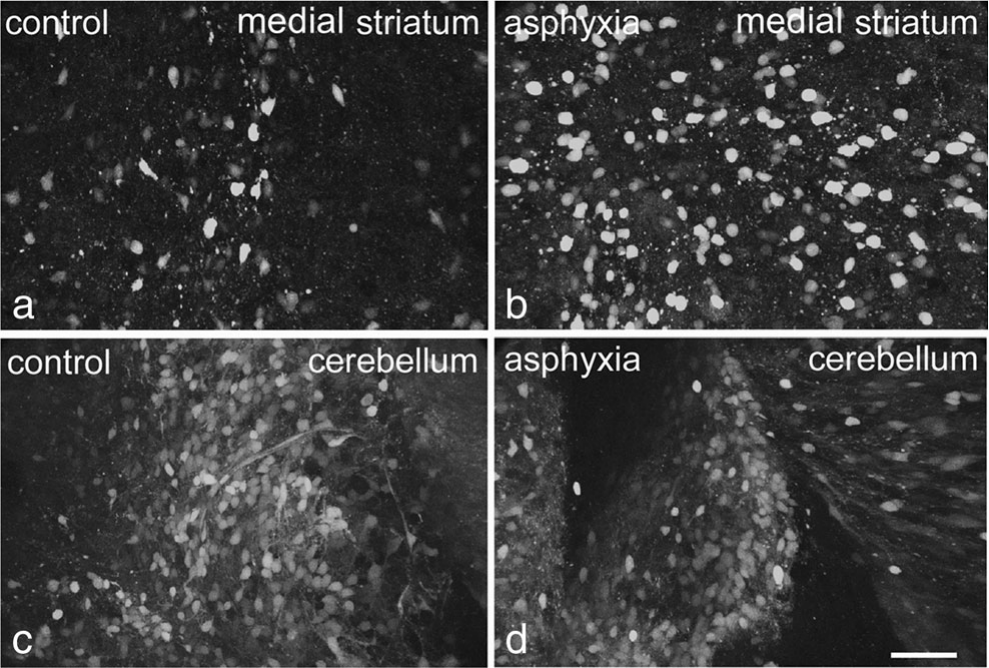
DAF-2 fluorescence in tissue slices from medial striatum (**a**, **b**) and cerebellum (**c**, **d**) of a control (**a**, **c**) and asphyctic rat (**b**, **d**) at postnatal day 8. Images were taken throughout the slice (300 μm) with a confocal laser scanning microscope and combined into one image per area using an image analysis system. *Scale bar* is 50 μm for all photographs

A bright fluorescent signal could be observed in the striatum and cerebellum of all pups after loading with DAF-2/DA. The fluorescent signal was localized intracellular in neuron-like cells and their proximal dendrites and in addition in vascular structures. Fluorescence in DAF-2 loaded slices of control rat pups was clearly visible throughout the entire striatum, predominantly in the dorso-medial part. Within the cerebellum, DAF-2/T fluorescence was visible throughout the molecular and granular cell layer (Fig. 1). The cerebellum had higher NO/peroxynitrite levels than the striatum at all time-points after birth. In control rat pups, the intensity of the DAF-2/T fluorescent signal in the striatum was higher at P5 and P8 than that at P12 (see Table 1). In the PA group, the intensity of the fluorescent signal was greater than in the control group at P5 and P8, but not at P12 (see Table 1). There was no apparent difference in fluorescence pattern or their intensity in the cerebellum between control and PA rats at P5, P8 or P12 (Fig. 1). The fluorescent signal was however less intense at P12 than at P5 or P8.

**Table 1:** Summary of the experiments performed with rat striatal slices at postnatal day P5, P8 and P12. Numbers represent the number of experiments. Per experiment, at least three slices throughout the striatum (300 μm) were studied. For image scoring references, see Fig. 3 (absence in 3E, weak in 3C, D, F strong in 3A, B, very strong in 3 G, H)

Age		Control			Asphyxia		
		Basal	N^G^-L-Nitro-arginine	SNP	Basal	N^G^-L-Nitro-arginine	SNP
P5	Absence DAF	0	1	0	0	2	0
	Weak DAF	4	4	0	0	4	0
	Strong DAF	4	0	5	6	1	3
	Very strong DAF	0	0	2	2	0	4
P8	Absence DAF	0	2	0	0	1	0
	Weak DAF	4	4	1	1	3	0
	Strong DAF	3	0	3	4	3	6
	Very strong DAF	0	0	2	3	0	1
P12	Absence DAF	1	3	0	0	5	0
	Weak DAF	5	2	0	6	1	0
	Strong DAF	0	0	6	1	0	1
	Very strong DAF	0	0	0	0	0	5

**Fig. 2:**
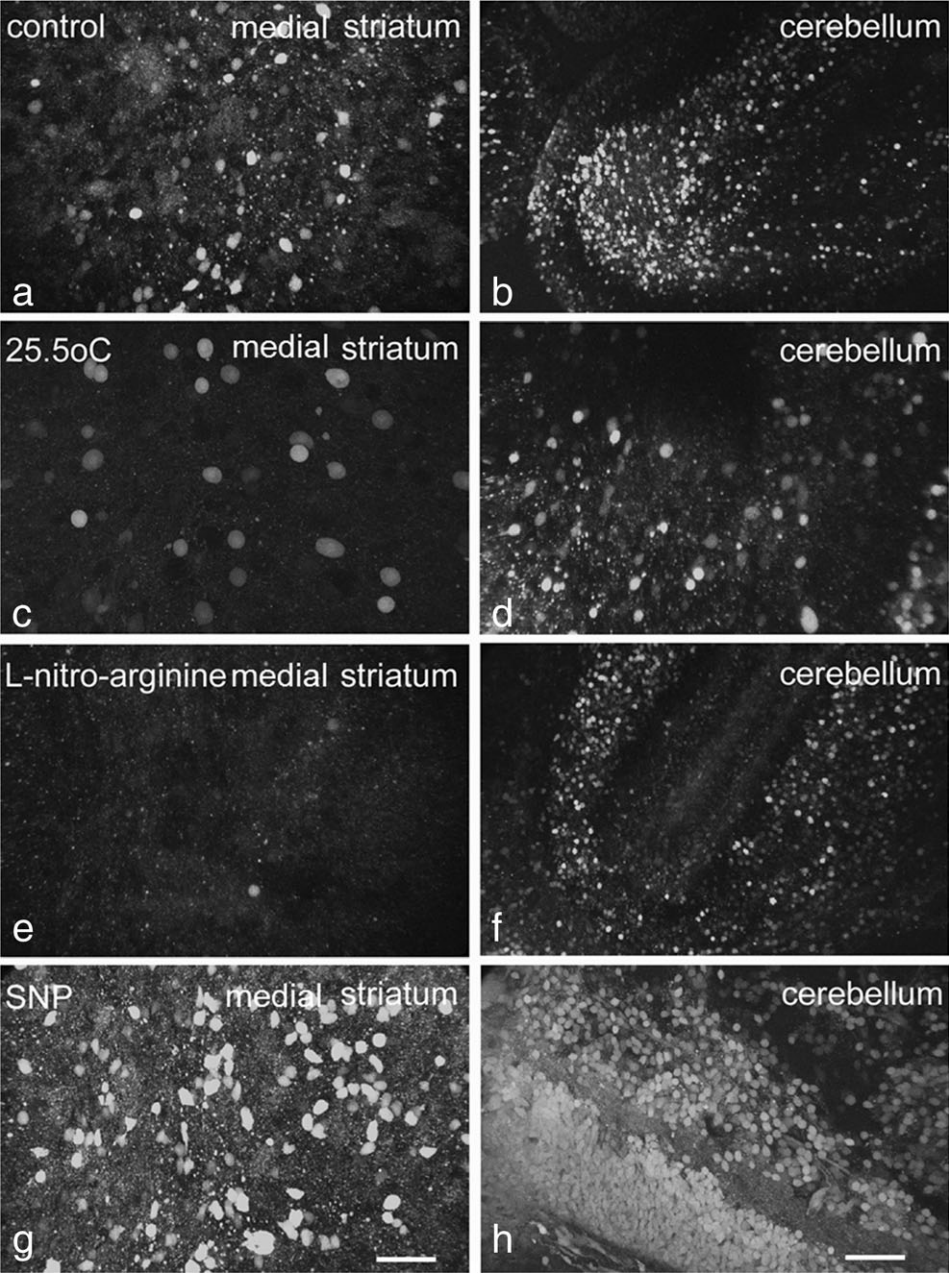
DAF-2 fluorescence in tissue slices of the medial striatum (**a**) and cerebellum (**b**) of a control rat incubated in the presence of 1 mM IBMX at 35.5°C for 45 min and **c**, **d** in the presence of 1 mM IBMX at 25.5°C for 45 min; **e**, **f** incubated in the presence of 0.1 mM *N* ^*G*^-nitro-L-arginine at 35.5°C for 45 min; and **g**, **h** incubated in the presence of 0.1 mM SNP at 35.5°C for 45 min. Hypothermia or incubation with *N* ^*G*^-nitro-L-arginine led to a decrease in fluorescent signal in both the striatum (**c**, **e**) and cerebellum (**d**, **f**). Pre-incubation with SNP led to a strong increase of the fluorescent signal in both the striatum (**g**) and cerebellum (**h**). *Scale bar* is 50 μm for all photographs

Pre-incubation with the NO donor SNP led to a major increase in the intensity and density of the DAF-2/T fluorescent signal in both striatum and cerebellum. Background levels were more intense after pre-incubation with SNP, and almost all cells showed an extremely bright diffuse fluorescence (see Fig. 2 and Table 1). Pre-incubation with *N* ^*G*^-L-nitro-arginine (0.1 mM, a NOS inhibitor) slightly suppressed the DAF-2/T fluorescent signal at P5, P8 and P12 in both striatum and cerebellum of PA rats at 35.5°C. Lowering the incubation temperature to 25.5°C drastically lowered the signal.

**Fig. 3:**
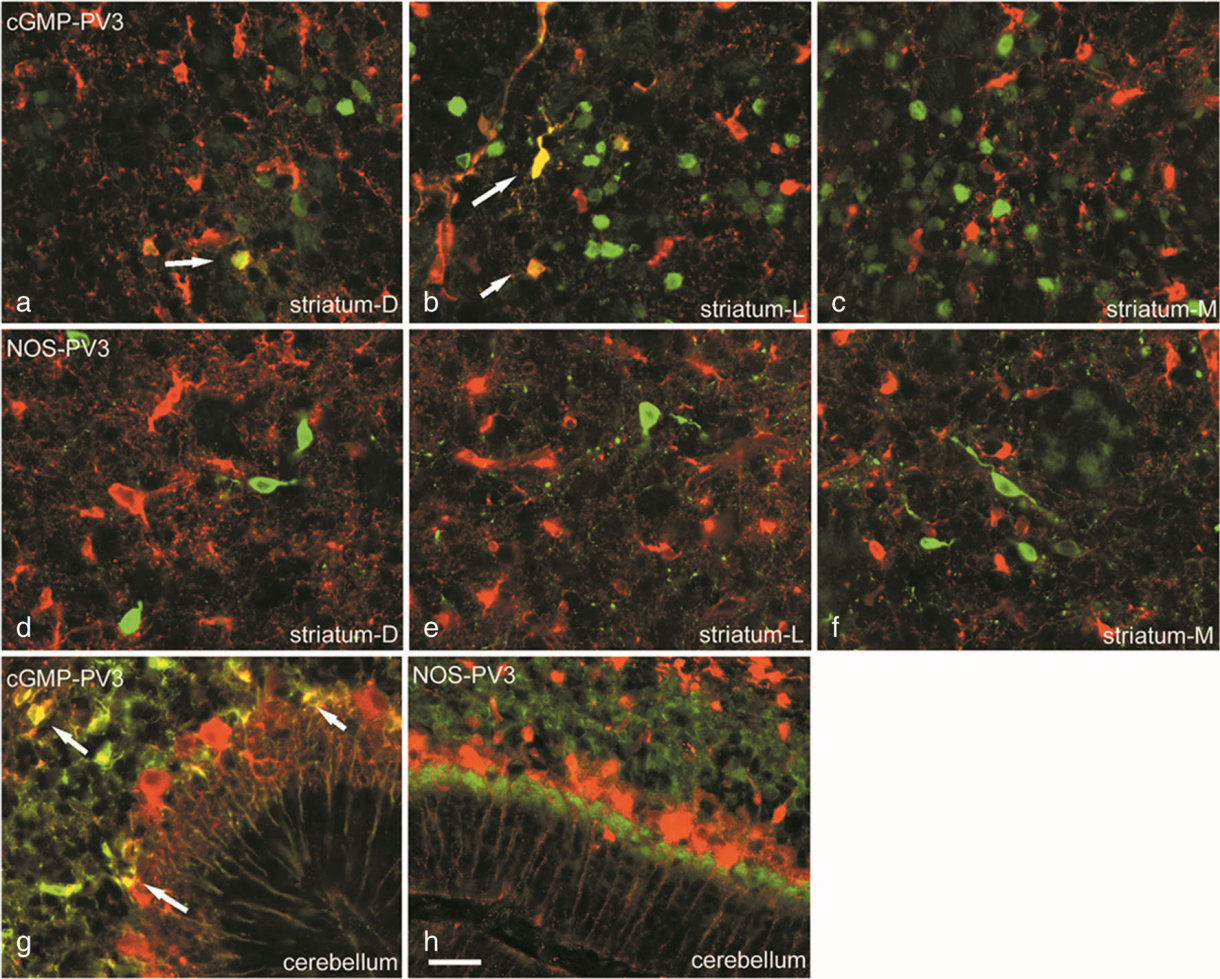
Double labelling of parvalbumin (PV-3, in green) and NO production markers, cGMP or nNOS (in *red*). *Yellow cells* indicate double labelling and thus co-localization. Double labelling of PV-3 and cGMP in the dorsal (**a**), lateral (**b**) and medial striatum (**c**) and cerebellum (**g**). Double labelling of PV-3 and nNOS in the dorsal (**e**), lateral (**f**) and medial (**g**) striatum and cerebellum (**h**). *Scale bar* is 50 μm

Data on NO-mediated cGMP synthesis were obtained from slices incubated with 1 mM IBMX to inhibit PDE activity. Incubation of the rat slices at P5, P8 and P12 with 1 mM IBMX resulted in intense cGMP immunoreactivity (cGMP-IR) in cell bodies and fibres in the striatum and cerebellum. Pre-incubation with 0.1 mM NG-L-nitro-arginine abolished the cGMP signal, indicating that this NOS inhibitor abolished NOS activity, and as a consequence NO production and cGMP synthesis. Incubation of the slices with SNP (0.1 mM, a NO donor) resulted in an increase of cGMP-IR in cells and fibres in both the striatum and cerebellum of both groups. The PA group had increased cGMP-IR in cells and fibres throughout the striatum compared to the control group at P5 and P8 (*P* < 0.05), but not at P12 (*P* > 0.05). nNOS-IR was observed in cell bodies and varicose fibres in grey and white matter of the striatum and cerebellum. Co-localization between cGMP and parvalbumin, but not between nNOS and parvalbumin, was found in cells and fibres in the striatum and cerebellum at P5, P8 and P12 (see Fig. 3). There was no detectable difference in nNOS-IR between control and asphyctic pups in the striatum or cerebellum. The results of the present study show that PA markedly increases NO/peroxynitrite during first postnatal week (P5 and P8, but not at P12), in the rat striatum. NO production in the cerebellum showed a similar trend, but no significant differences were observed between control and PA groups.

### Caspase-3 Distribution and Activity and Astrogliosis in the Rat Asphyctic Stratum and Cerebellum

Caspase-3 positive cell profiles were visible in both the grey and white matter of striatum and cerebellum of control and asphyctic pups during the first week of postnatal life. In the cerebellum, caspase-3-positive cells were found within white matter and in the granular cell layer at P2 and P5. At P8, caspase-3 positive cells were mainly observed within the granular cell layer. The caspase-3 immunohistochemistry was similar between the control and PA groups.

**Fig. 4:**
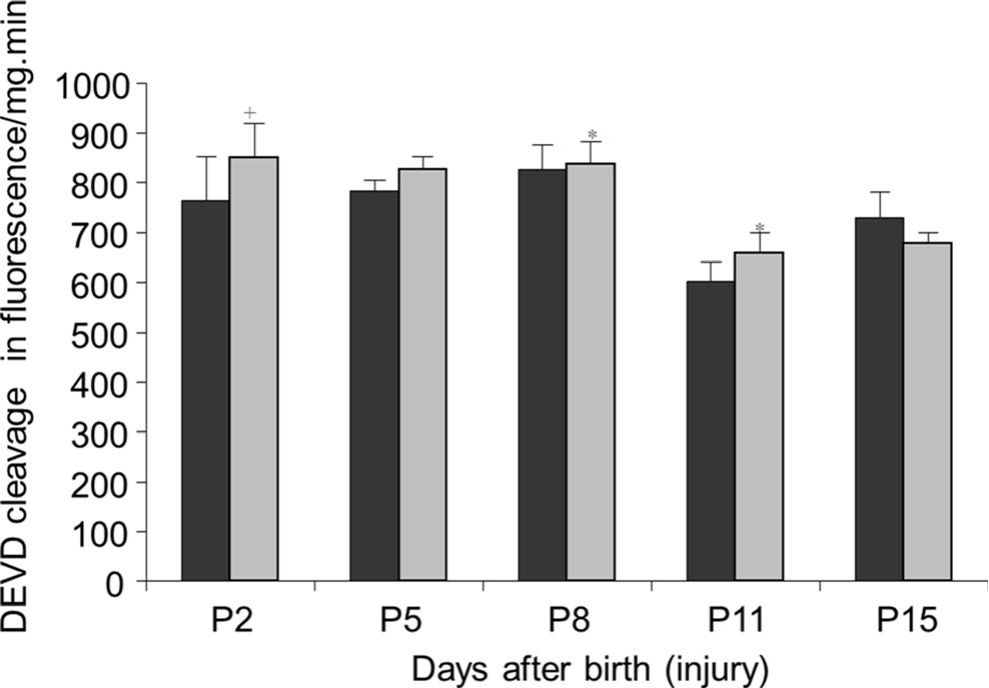
Caspase-3-like activity (DEVD cleavage, expressed in cleaved AMC fluorescence per mg wet weight per minute) within the cerebellum of control (*in black*) and asphyctic (*in grey*) rats during the first 15 days after birth. The caspase-3-like activity after global asphyxia was compared using a two-way ANOVA with a Bonferroni correction for repeated measures. (*Plus sign*) *P* = 0.06, (*asterisk*) *P* < 0.05

**Fig. 5:**
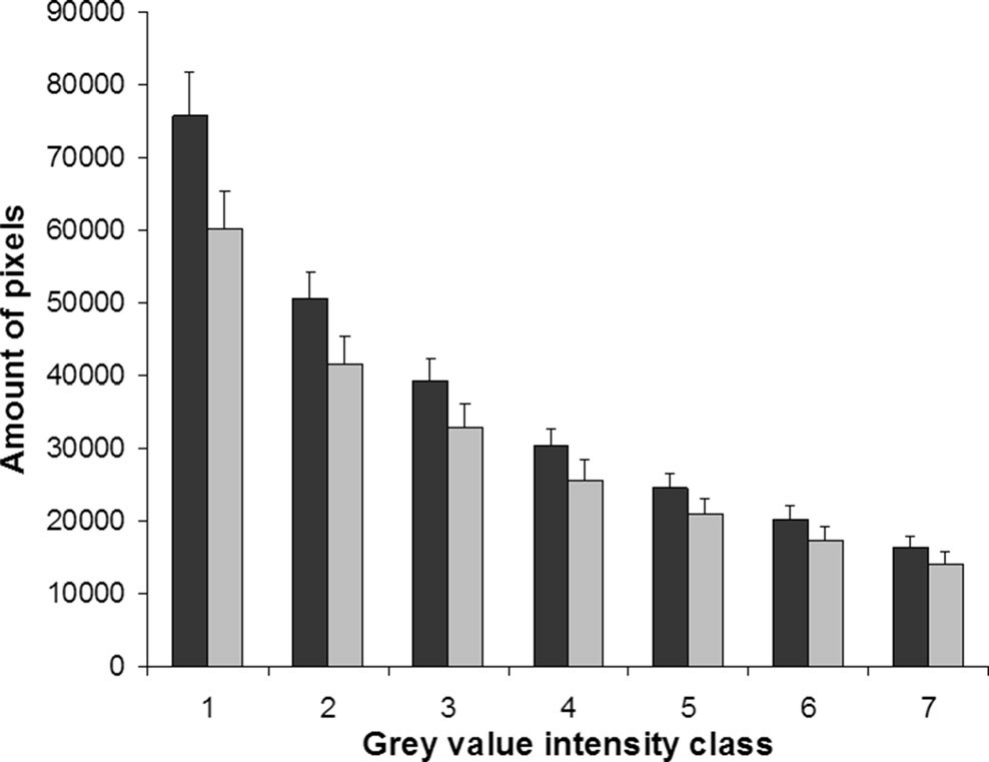
Histogram of division of pixels over intensity classes of GFAP immunoreactivity in striata of control (shown in *black*, *n* = 5) and asphyctic rats (shown in *grey*, *n* = 5), at P12. Immunofluorescence intensity was converted to grey values divided over 16 classes. The results of classes 10–16 are shown as almost no pixels were present in other classes. There was no difference in distribution or amount of pixels per grey value intensity class between the groups. Data are expressed as mean ± SEM

To assess the time course of caspase-3-like activity during normal development and after PA, we measured DEVD cleavage in homogenates of cerebellum of control and PA rats during the first 15 days of postnatal life. The results of DEVD cleavage activity are shown in Fig. 4. The pattern of DEVD cleavage in the cerebellum was stable during the first week of life, followed by a decrease at P11 (P8 vs. P11; *P* < 0.01) and an increase at P15. The time course of DEVD cleavage activity was not different between control and asphyctic group and cerebellum, but the DEVD cleavage was moderately higher in the PA group at P8 and P11 (PA vs. control; *P* < 0.05) and lower at P15 (not significant). This corresponds to the cerebellar DAF-2/T fluorescence which also did not show large variation between groups and which was higher at P5 and P8 than that at later time-points. Overall, the regional distribution and timing of apoptosis in the striatum and cerebellum were similar between the PA and control groups in the first week, although there were modest relative increases in caspase-3 cleavage in the PA group up until P11. Astrogliosis also did not vary significantly between the PA and control groups (Fig. 5).

## Discussion

### Regional Differences in NO Production and Apoptosis

In the striatum, we observed a supraphysiological increase in NO/peroxynitrite and cGMP, which was not present in the cerebellum. The striatal NO production was not accompanied by an increase in nNOS expression. This is consistent with observations that iNOS, rather than nNOS or eNOS, is upregulated after asphyxia. iNOS greatly increases NO concentrations, which leads to peroxynitrite formation (Ikeno et al. 2000). Another group showed decreased striatal nNOS-positive cells in organotypic cell cultures made 3 days after PA, accompanied by PA-related increases in nNOS-expressing cells in the substantia nigra, a region that normally has lower nNOS activity than the striatum (Klawitter et al. 2006; Klawitter et al. 2007). In the cerebellum, the coupling between increased neuronal activity and local blood flow relies almost exclusively on NO (Rancillac et al. 2006). Thus, under physiological conditions, the cerebellum has the highest NOS activity and the highest concentration of glutamate and aspartate in the brain (Blanco et al. 2010). NO/peroxynitrite production in the striatum and cerebellum was higher on P5 and P8 than on P12. The effect of PA on NO was thus maximal during first postnatal week. The NO/peroxynitrite production was higher in the cerebellum than that in the striatum at all time-points, but PA did not cause a supraphysiologic increase on top of the NO production seen in the control groups.

In both the striatum and cerebellum, there are sub-populations of neurons that would be vulnerable to supraphysiologic NO production—and relatively resistant neurons that express nNOS. Ninety-five percent of the striatal neurons are GABAergic projection neurons (medium spiny neurons), and the remaining 5% is made up by interneurons. The interneurons are divided into tonically active cholinergic neurons, fast-spiking GABAergic interneurons, which express parvalbumin, and low-threshold spiking interneurons, which express somatostatin, neuropeptide Y and nNOS. The low-threshold spiking interneurons are the primary source of striatal nNOS (Lenz and Lobo 2013; Liljeholm and O’Doherty 2012). nNOS is maximally expressed in the striatal regions where immature NMDA receptors are expressed (Black et al. 1995). Upon excessive stimulation of the NMDA receptors during PA, peroxynitrite is increased in the region (Ferriero et al. 1990; McQuillen and Ferriero 2004). The NO-producing interneurons are resistant to PA and NMDA-mediated excitotoxicity. However, the nearby striatal projection neurons are vulnerable to increased peroxynitrite formation. This selective vulnerability of the striatal projection neurons may result from a bystander effect attributable to their proximity to the enriched population of nNOS-expressing interneurons neurons (Ehrlich 2012; McQuillen and Ferriero 2004; Titomanlio et al. 2015). The relative resistance of the low-threshold spiking nNOS interneurons is echoed by our observations. The nNOS immunoreactivity did not change, and thus presumably PA did not alter the amount of nNOS-expressing interneurons in the striatum. However, NO/peroxynitrite and cGMP increased in the striatum and cGMP partially co-localized with parvalbumin neurons (Fig. 3), which indicates that there was more NO available to the parvalbuminergic neurons. Parvalbumin fast-spiking and cholinergic interneurons in the striatum have demonstrated vulnerability to PA (de Vente et al. 2000; Kohlhauser et al. 1999; Van de Berg et al. 2002).

In the cerebellum, nNOS is expressed by both the mature granule neurons and the molecular layer interneurons (basket and stellar cells) (Contestabile 2012) and these cells are thus relatively more resistant to NO/peroxynitrite toxicity. The Purkinje cell population (which express parvalbumin, but not nNOS) is sensitive to peroxynitrite, but these cells also need a small amount of NO to survive (Oldreive et al. 2012). During normal postnatal development, the expression and activity of cerebellar nNOS increases slowly throughout the first week. The granule neurons only show detectable NOS reactivity after migrating to the internal granular layer (Contestabile 2012). We observed NO/peroxynitrite production throughout the molecular and granular layer of the cerebellum in both control and PA rats. nNOS expression and cGMP production occurred throughout the cerebellum in both groups at 35.5°C. Lowering the temperature by 10°C drastically lowered NO production. This correlates with the clinical observations that selective head cooling is beneficial after PA (Guillet et al. 2012).

We only observed changes in the cerebellar caspase-3-like activity in the second week of postnatal life in both control and asphyctic rats. The pattern of caspase-3-like activity in the cerebellum is unique in that it declines after the first week of postnatal life, but increases again afterwards (P15). There was a modest increase in caspase-3 activity in the asphyctic groups up until P11, but overall, the patterns of caspase-3-like activity (and distribution of caspase-3 immunoreactive cells) was similar between groups and probably reflects physiological cell death. Apoptotic cell death is crucial for the normal development of the CNS and occurs in all brain areas during foetal and postnatal life (Devoto et al. 2013).

Although the striatum as a whole is susceptible to PA, not all striatal neurons are affected equally after PA. The nNOS-expressing interneurons were spared in our model. This is also seen in chronic degenerative diseases of the striatum that lead difficulties in motor control, like Huntington’s disease. In Huntington’s, the striatal neurons that do not release NO degenerate during the disease course, but the nNOS-producing interneurons are spared. The striatal projection neurons that form cortico-striatal network are particularly sensitive to glutamate-induced excitotoxicity and NO release (Canzoniero et al. 2014; Ehrlich 2012).

Previous NO-related research in the same model of asphyxia showed that increased striatal cGMP production is still evident at P10 (Loidl et al. 1998). However, directly after PA, when the foetal circulation and metabolism is still maintained, there were no apparent changes in the activity or transcription of nNOS (Lubec et al. 1999) or inducible NOS (Calamandrei et al. 2004). Early intervention in NO production could have long-term therapeutic effects. At 6 months, PA-related increases in striatal NOS activity were still apparent. The NOS-containing neurons had ultra-structural changes including cytomegaly, and the surrounding neurons showed increased degeneration (Capani et al. 1997). In the same regions, neurons that express nicotinamide adenine dinucleotide phosphate diaphorase (NADPH-d)—a marker of NO synthesis—had similar cytomegalic morphology. The gross amount of NADPH-d expressing neurons was not altered by PA (Loidl et al. 1997). The results of the timing data suggest that the optimal therapeutic window for a NO-targeted therapy in the preterm infant would be in the first week after PA. Overall, our results show that reducing NO production could be a potential therapy to reduce striatal damage. The protective effects of NO-reduction may be limited to the regions with supraphysiological increases in NO.

## Conclusion

From these observations, we conclude that PA has a greater impact on NO/peroxynitrite production in the striatum than that in the cerebellum. Although NO/peroxynitrite was observed throughout the cerebellum, this can be ascribed to the high nNOS activity of the region. The greatest increase in NO production occurred during the first week, although it was previously reported that this change is not evident directly after PA. A therapeutic window exists within the first week after PA, during which inhibitors of NO production would likely have their best therapeutic effect in the preterm brain. Although this type of intervention would likely only show regional benefits in the brain regions with lower initial NO production, attenuating striatal neuron loss could have a motor-benefit after PA. Astrocytes were not notably increased by PA, thus the striatal NO/peroxynitrite increase is likely independent of infiltration by inflammatory cells. Targeting inflammation alone is likely to be ineffective after PA, and alternate pathways should be considered.

## Electronic supplementary material

Fig. S1 An overview of the experimental design (GIF 188 kb)

High Resolution Image (TIFF 100 kb)
