# Supplementary figures and images for: Nitric Oxide Production in the Striatum and Cerebellum of a Rat Model of Preterm Global Perinatal Asphyxia
Source: Neurotox Res. 2017 Jan 21;31(3):400–9. doi: 10.1007/s12640-017-9700-6 (PMC5360831; doi:10.1007/s12640-017-9700-6)

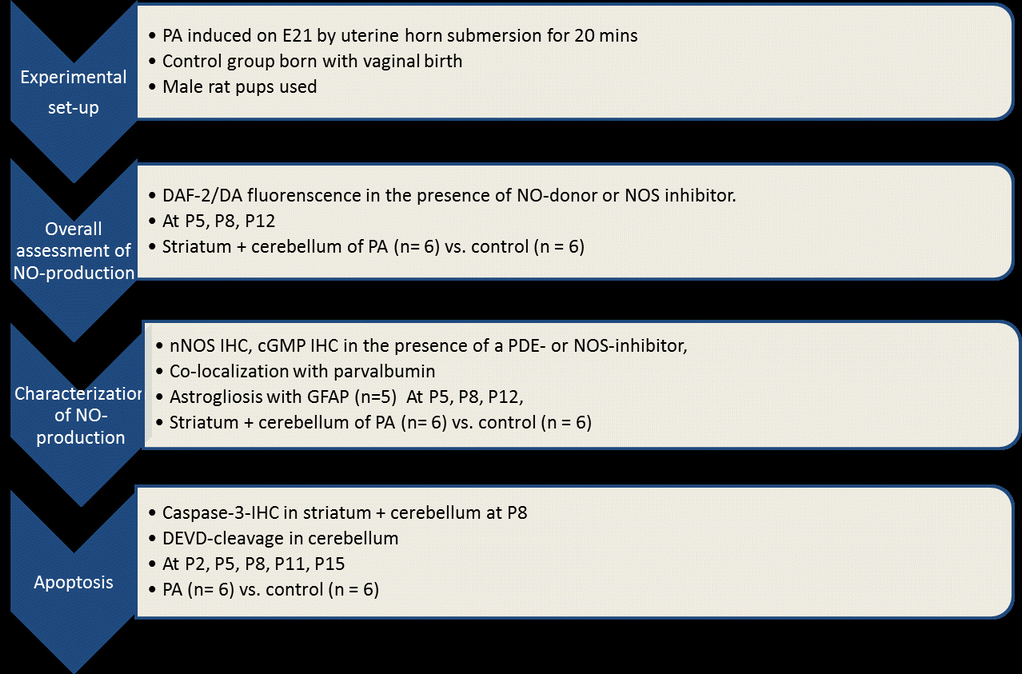

Supplement: Supplementary file 1 — An overview of the experimental design (GIF 188 kb) [file 12640_2017_9700_Fig6_ESM.gif]

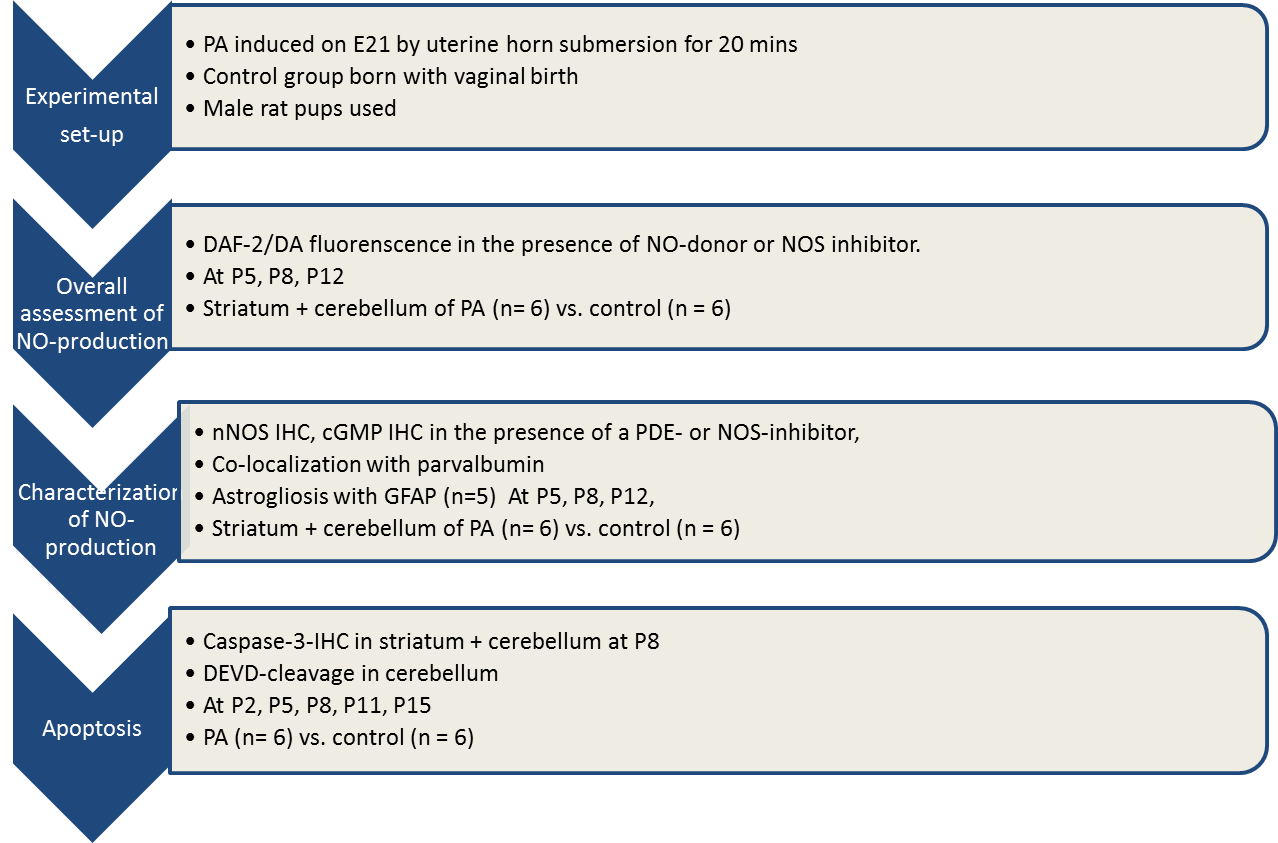

Supplement: Supplementary file 2 — High Resolution Image (TIFF 100 kb) [file 12640_2017_9700_MOESM1_ESM.tif]
